# Editorial: Vitamin D Binding Protein, Total and Free Vitamin D Levels in Different Physiological and Pathophysiological Conditions

**DOI:** 10.3389/fendo.2020.00040

**Published:** 2020-02-11

**Authors:** Zhongjian Xie, Xiangbing Wang, Daniel D. Bikle

**Affiliations:** ^1^Hunan Provincial Key Laboratory of Metabolic Bone Diseases, National Clinical Research Center for Metabolic Diseases, Department of Metabolism and Endocrinology, The Second Xiangya Hospital of Central South University, Changsha, China; ^2^Division of Endocrinology, Metabolism and Nutrition, Rutgers University-Robert Wood Johnson Medical School, New Brunswick, NJ, United States; ^3^Endocrine Unit, Veterans Affairs Medical Center, University of California, San Francisco, San Francisco, CA, United States

**Keywords:** vitamin D binding protein, total 25-hydroxyvitamin D, free 25-hydroxyvitamin D, 1,25-dihydroxyvitamin D, bioavailable 25-hydroxyvitamin D

Vitamin D binding protein (DBP) is a major plasma carrier for vitamin D and its metabolites. In recent years, there has been growing interest in understanding the physiological functions and attributes of DBP. The current issue is comprised of five review articles and two original research papers concerning the physiology of DBP and its role in different disorders.

Poor vitamin D status is highly prevalent in many different countries (1–4), but the exact definition of vitamin D status is controversial. The plasma concentration of total 25-hydroxyvitamin D [25(OH)D] is currently used as an indicator of vitamin D status. In the past decades, however, there has been argument as to whether just measuring total 25(OH)D is appropriate for the assessment of vitamin D status in different physiological and pathophysiological conditions (5, 6). About 85% of the total circulating 25(OH)D is bound to DBP, and 15% is bound to albumin. About 0.03% of 25(OH)D circulates in free form. Since 25(OH)D is weakly bound to albumin and dissociates from it during tissue perfusion, the sum of the free and the albumin-bound 25(OH)D represents the bioavailable 25(OH)D, which may be readily available for metabolic function. In contrast, the DBP-bound vitamin D is relatively unavailable to target tissue, with the exception of a few tissues such as the kidney that express a megalin/cubilin transport system for DBP-bound 25(OH)D. The concept that it is the free hormone and not the DBP-bound hormone that enters cells is known as the free hormone hypothesis. In the review by Bikle and Schwartz it is highlighted that the DBP level is regulated by estrogen, glucocorticoids, and inflammatory cytokines but not by vitamin D itself, and therefore, these regulators would affect levels of total 25(OH)D. The review by Bikle and Schwartz focuses on the biological importance of DBP with emphasis on its regulation of total and free vitamin D metabolite levels in various clinical conditions. They also point out that attempts to calculate the free level using affinity constants generated in a normal individual along with measurement of DBP and total 25(OH)D have not accurately reflected directly measured free levels in a number of clinical conditions. The authors examine the impact of different clinical conditions as well as different DBP alleles on the relationship between total and free 25(OH)D, using only data in which the free 25(OH)D level was directly measured. Following their previous review (7), the review by Chun et al. discussed a number of important questions including the following. Is the total 25(OH)D (bound plus free) or the unbound free 25(OH)D the crucial determinant of the non-classical actions of vitamin D? While DBP-bound 25(OH)D is important for renal handling of 25(OH)D and endocrine synthesis of 1,25(OH)_2_D, how does DBP impact extra-renal synthesis of 1,25(OH)_2_D and subsequent 1,25(OH)_2_D actions? Are there pathophysiological contexts where total 25(OH)D and free 25(OH)D would diverge in value as a marker of vitamin D status? This review aims to introduce the concept of free 25(OH)D and the molecular biology and biochemistry of vitamin D and DBP, which provides the context for free 25(OH)D, and surveys *in vitro*, animal, and human studies taking free 25(OH)D into consideration.

Low DBP levels in patients with primary hyperparathyroidism (PHPT) were first reported in 2013 by Wang's group (8) and confirmed by Battista et al. (9). In the paper by Wang et al., the authors recruited 75 patients with PHPT and 75 healthy control subjects. In addition, 25 PHPT patients underwent parathyroidectomy and had a 3-month follow up visit. The results showed that serum DBP levels were lower in patients with PHPT but that parathyroidectomy restored DBP levels. Lower DBP levels may be one of the contributing factors of low total 25(OH)D level in PHPT patients, and the total 25(OH)D levels might not reflect true vitamin D status in patients with PHPT.

In the comprehensive review by Bouillon et al. it was noted that DBP was originally discovered as a highly polymorphic protein useful for population studies and originally called Group-specific Component (GC). It is now known that DBP and GC are the same protein and appeared early in the evolution of vertebrates. DBP is genetically the oldest member of the albuminoid family (which includes albumin, α-fetoprotein, and afamin, all involved in the transport of fatty acids or hormones). DBP has a single binding site for all vitamin D metabolites with a high affinity for 25(OH)D, thereby creating a large pool of circulating 25(OH)D, which prevents rapid vitamin D deficiency. The review also highlighted the roles of DBP in preventing the urinary loss of 25(OH)D and the formation of polymeric actin fibrils in the circulation after tissue damage. DBP also plays a minor role in transporting fatty acids. Based on the fact that the total concentrations of 25(OH)D and 1,25(OH)_2_D in DBP null mice or humans are extremely low but calcium and bone homeostasis remain normal, the “free hormone hypothesis” appears to apply to the vitamin D hormones, 25(OH)D or 1,25(OH)_2_D, as it does to other steroid hormones and thyroid hormone.

Vitamin D is important for bone health but may also have extra-skeletal effects. Cunningham et al. examined vitamin D metabolites in serum samples from age- and weight-matched women with and without PCOS and reported results in their paper. The authors found that 25-hydroxy-3epi-Vitamin D_3_, 25-hydroxyvitamin D_2_, 25-hydroxyvitamin D_3_, and 24,25-dihydroxyvitamin D_3_, but not 1,25-dihydroxyvitamin D_3_ [1,25(OH)_2_D_3_], were associated with embryo parameters. The data suggest that vitamin D metabolites other than 1,25(OH)_2_D_3_ are important in fertility. Kew, in his review assesses the fundamental role of DBP in neutrophilic inflammation and injury. As highlighted by Kew, DBP induces selective recruitment of neutrophils. DBP is also an extracellular scavenger for actin released from damaged/dead cells, and formation of DBP-actin complexes is an immediate host response to tissue injury. DBP bound to G-actin functions as an indirect but essential cofactor for neutrophil migration.

Vitamin D and DBP have immunological effects and may be important in the development of type 1 diabetes (T1DM). Moreover, low total 25(OH)D levels are associated with the development of type 2 diabetes (T2DM). However, there are no convincing data showing that vitamin D supplementation has an effect on the prevention of T2DM (10). The review by Jorde discusses the relations between DBP and total and free 25(OH)D in T1DM and T2DM.

## Author Contributions

All authors listed have made a substantial, direct and intellectual contribution to the work, and approved it for publication.

### Conflict of Interest

The authors declare that the research was conducted in the absence of any commercial or financial relationships that could be construed as a potential conflict of interest.
